# GP-9s Are Ubiquitous Proteins Unlikely Involved in Olfactory Mediation of Social Organization in the Red Imported Fire Ant, *Solenopsis invicta*

**DOI:** 10.1371/journal.pone.0003762

**Published:** 2008-11-19

**Authors:** Walter S. Leal, Yuko Ishida

**Affiliations:** Honorary Maeda-Duffey Laboratory, Department of Entomology, University of California Davis, Davis, California, United States of America; Cairo University, Egypt

## Abstract

The red imported fire ant (RIFA), *Solenopsis invicta*, is an invasive species, accidentally introduced in the United States that can cause painful (sometimes life-threatening) stings to human, pets, and livestock. Their colonies have two social forms: monogyne and polygyne that have a single and multiple functional queens, respectively. A major gene (*Gp-9*), identified as a putative pheromone-binding protein on the basis of a modest amino acid sequence identity, has been suggested to influence the expression of colony social organization. Monogyne queens are reported to possess only the *GP-9B* alleles, whereas polygyne queens possess both *GP-9B* and *GP-9b*. Thus, both social forms are reported to express GP-9B, with GP-9b being a marker expressed in polygynes but it is absent in monogynes. Here, we report two types of polygyne colonies, one that does not express GP-9b (monogyne-like) and the other expressing both proteins, GP-9B and GP-9b. Given their expression pattern, GP-9s are hemolymph proteins, which are more likely to be involved in the transport of lipids and small ligands within the homocoel. GP-9B existed in two forms, one of them is phosphorylated. The helical-rich content of the protein resembles the secondary structures of a beetle hemolymph protein and moth pheromone-binding proteins. An olfactory role is unlikely given the lack of specific expression in the sensillar lymph. In marked contrast to GP-9s, a chemosensory protein, SinvCSP, is demonstrated to be specifically expressed in the antennae. Within the antennae, expression of SinvCSP is restricted to the last two segments, which are known to house olfactory sensilla.

## Introduction

The red imported fire ant (RIFA), *Solenopsis invicta* Buren (synonym of *S. wagneri* Santschi), is an invasive species native to South America, which was accidentally introduced in the United States through the port of Mobile, AL, sometime between 1933 and 1945 [1], probably even earlier (http://www.invasivespeciesinfo.gov/animals/rifa.shtml). RIFA is now widespread in the Southeastern United States and has been introduced into Southern California (http://www.aphis.usda.gov/plant_health/plant_pest_info/fireants/downloads/fireant.pdf). This fire ant has two social forms. Monogyne colonies are those with a single functional queen, which may start by a single or few queens but only one survives the battle after the first workers appear. Polygyne colonies, on the other hand, have at least two functional queens and sometimes as many as twenty thousand. While polygyne societies are known to accept foreign queens of the monogyne- and polygyne-type, monogyne colonies may accept polygyne queens, but kill encroaching monogyne-type queens [2]. This colony social organization has been associated with a gene (*Gp-9*), which has two electrophoretically detectable alleles in introduced populations in the USA, with the *B* allele being the only allele found in the monogyne form, and the *b* allele occurring together with the *B* allele in the polygyne form [3]. Krieger and Ross [4] isolated GP-9s from the thoraces of monogyne and polygyne queens, cloned the genes encoding GP-9B and GP-9b, and based on modest amino acid sequence assigned them as putative pheromone-binding proteins (PBPs), which they suggested regulate social behavior in this fire ant. While polygyne expresses both GP-9B and GP-9b, monogyne queens expressed only GP-9B [4]. On the other hand, Fletcher and Blum [5] and Vander Meer and Alonso [6] elegantly demonstrated that worker aggression is mediated by a queen-produced recognition primer pheromone. While introduction of newly-mated queen intruders into queenright monogyne colony residents resulted in immediate investigation and eventual alarm and attack, queenless workers often aggregated around and walked over intruders [6]. The apparent conundrum of monogyne workers devoid of GP-9b perceiving a queen-produced recognition primer pheromone (with the implication that polygyne workers expressing GP-9B and GP-9b are anosmic to this pheromone) prompted us to revisit the putative role of GP-9s. Here, we report (1) that polygyne workers express one or two forms of GP-9s, which are demonstrated to be general hemolymph proteins unlikely involved in olfaction, and (2) on the identification of a chemosensory protein specifically expressed in olfactory tissues located on the terminal segments (club) of the antennae that house olfactory sensilla.

## Results and Discussion

### Isolation of GP-9B from Polygyne Workers

Since GP-9B and GP-9b were previously isolated from thorax extracts of queens [4] but queens are emitters and workers are the receivers of the recognition primer pheromone [5], [6], we aimed first at re-isolating these proteins from workers and investigating their potential role in olfaction. These general proteins from RIFA, GP0-8 [7] and GP-9 [3], have been identified from horizontal starch gels and gel profiles were not documented in the literature. Thus, we analyzed RIFA extracts with native polyacrylamide gels (PAGE), which provides more consistent and reproducible profiles for future references.

**Figure 1:**
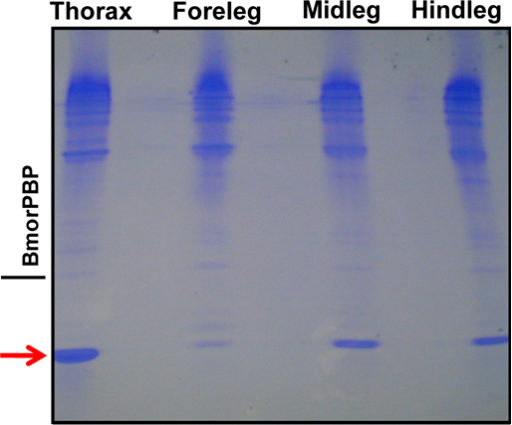
Analysis of proteins extracted from the thoraces and legs of polygyne workers collected in Riverside, CA. Protein extracts were separated on a 15% native PAGE and stained with Coomassie Brilliant Blue (CBB). A fast migrating protein (highlighted with a red arrow) was later identified as one of the GP-9s previously isolated from the thorax of RIFA queens [4]. The pheromone-binding protein from the silkworm moth, *Bombyx mori*, BmorPBP [8] was used as a reference. Each lane was loaded with 100 workers-equivalent.

**Figure 2:**
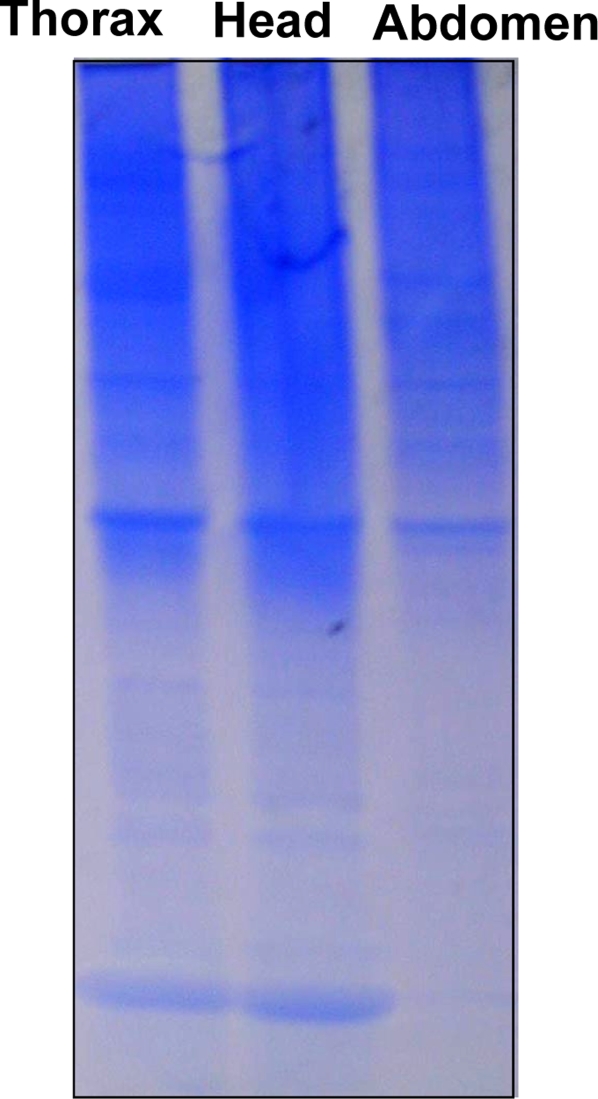
Gel electrophoresis of extracts from three body parts of workers collected in Riverside, CA. Proteins were separated on a 20% native gel, with each lane loaded with 100 workers-equivalent. The intensity of the bands in the upper part of the gel suggest that nearly equal amounts of the extracts were loaded, although the fast migrating band in the extract from the abdomen is almost below the detection limit of CBB.

**Figure 3:**
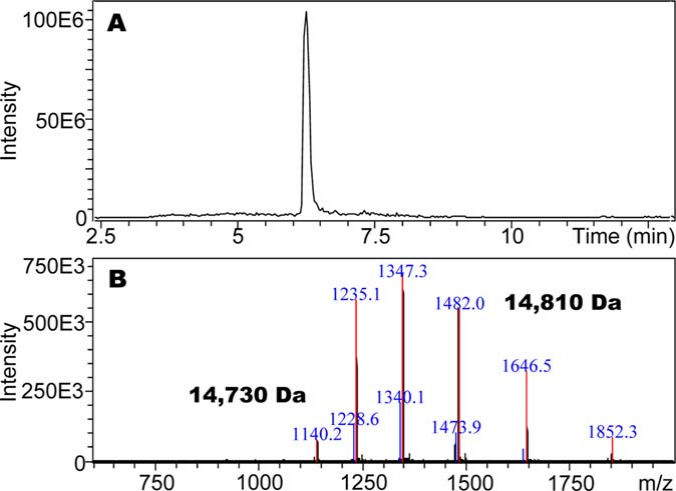
Mass spectral data for a sample of the first migrating band after purification. (A) HPLC trace showing a single peak, (B) MS profile suggesting that two proteins co-eluted. The charge envelopes were deconvoluted to give molecular masses of 14,730 Da (minor protein) and 14,810 Da (major protein). The molecular mass of the minor protein matches that of GP-9B considering the formation of the three disulfide bridges.

**Figure 4:**
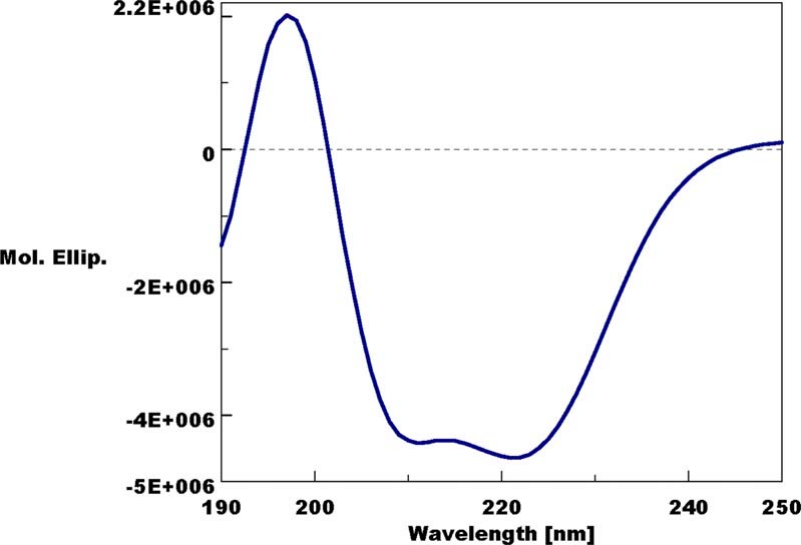
Far-UV CD spectrum of a sample of the fast migrating protein after chromatographic purification. The two minima at ca. 210 and 222 nm indicate a helical-rich protein. The sample used for this measurement was confirmed by LC-ESI/MS to have a single HPLC and a single MS peak that gave the molecular mass of 14,810 Da.

Native-PAGE analysis of various tissue extracts (thorax, foreleg, midleg, and hindleg) from polygyne workers collected in Riverside, CA showed a distinct band at the bottom of the gel, which migrated faster than a standard, the pheromone-binding protein (PBP) from the silkworm moth, *Bombyx mori*, BmorPBP [8] (Fig. 1). We compared the protein profiles from thorax extract with those obtained from head and abdomen and noticed that the fast migrating band was again present in all extracts, with significantly reduced amounts being detected in the extract from abdomen (Fig. 2). After isolating the target band by electroblotting and subsequent amino acid sequencing employing Edman degradation, the N-terminal sequence of this major band was identified as Ser-Arg-Asp-Ser-Ala-Arg-Lys-Ile-Gly, which is identical to the N-termini of both GP-9B and GP-9b [4]. cDNA cloning from 28 independent clones suggested that this major band is a product of Gp-9B. To unambiguously correlate the cDNA data with the isolated protein band, we measured the molecular mass of the fast migrating protein by LC-ESI/MS. The protein isolated by ion exchange and gel filtration chromatography gave a single peak by liquid chromatography (Fig. 3A), but the mass spectral data indicated that two proteins with molecular masses of 14,810 and 14,730 Da co-eluted (Fig. 3B) in the same HPLC peak (Fig. 3A). Interestingly, the molecular mass of the minor protein in the HPLC peak matched the calculated molecular mass for GP-9B considering the formation of three disulfide bridges (calculated, 14,731 Da). Other fractions showed a single HPLC and MS profile for a single protein with molecular mass of 14,810 Da (data not shown). Analysis of a pure sample by far-UV circular dichroism (CD) data (Fig. 4) indicated that the 14,810 Da protein is an α-helix-rich protein, a structural feature of moth PBPs [8], [9] and a hemolymph protein from the mealworm beetle, *Tenebrio molitor*, (THP12) [10], [11]. We then reasoned that the difference in the observed and calculated molecular masses could be due to phosphorylation of GP-9B, in addition to formation of disulfide bridges. The latter is the only known posttranslational modification for PBPs and other odorant-binding proteins [12], [13]. When treated with alkaline phosphatase the two MS profiles (Fig. 3B) coalesced to a single MS profile identical to the minor peak of molecular mass 14,730 Da (Fig. 3B). Similar results were obtained with pure fractions of the protein with molecular mass of 14,810 Da (data not shown). Thus, we conclude that in marked contrast to the hitherto known pheromone-binding proteins, GP-9B undergoes phosphorylation and thus existed in two forms, phosphorylated GP-9B (pGP-9B, observed MM 14,810 Da) and dephosphorylated form (GP-9B, observed MM 14,730 Da). There are ten potential phosphorylation sites in GP-9B: Ser-4, 10, 23, 36, 67, 113, 130; Thr-103; Tyr-12, 71, 80 (NetPhos 2.0 Server, http://www.cbs.dtu.dk/services/NetPhos/). To determine the phosphorylated residue the protein was first treated with DTT and iodoacetamide and then digested with Lys-C. The reaction mixture was analyzed by HPLC and LC-ESI/MS. All peptides (larger than 7 amino acid residues) expected as reaction products were detected by LC-ESI/MS, except for the fragment 113-134 containing two potential phosphorylation sites. We postulated that the negative charges from phosphorylation made the mass detector “blind” to this peptide. We then separated the reaction mixture by preparative-HPLC and analyzed the collected fractions by both LC-ESI/MS and Edman degradation. That way we matched the molecular masses of the fragments with their N-terminal amino acid sequences. For example, the peptide generated by cleavage at Lys-7 and Lys-41 gave the molecular mass of 3,772 Da (calculated 3,771.7 Da, considering the formation of carbamidomethyl-cysteine-18) and the N-terminal sequence: Xxx-Gly-Ser-Gln-Tyr-Asp-Asn-Tyr-Ala thus confirming the identification of the fragment 8-41. Likewise, fragments 42-51, 52-62, 63-75, and 83-104 were identified. While smaller fragments (1-7, 76-82, 105-110 and 110-112) probably eluted with the solvent front, the fragment 113-134 gave no signal by MS signal, but N-terminal sequencing confirmed its assignment. The fragment contains two Ser residues, but only one of them, Ser-130, has high phosphorylation potential (NetPhos 2.0 Server, http://www.cbs.dtu.dk/services/NetPhos/). Ser-113 was ruled out not only on the basis of low phosphorylation potential, but also because it was not modified and thus detected in the N-terminal sequencing: Ser-Leu-Leu-Leu-Ala-Ala-Xxx-Ile-Leu. Therefore, we inferred that Ser-130 contained in this peptide fragment is the site of phosphorylation in pGP-9B.

**Figure 5:**
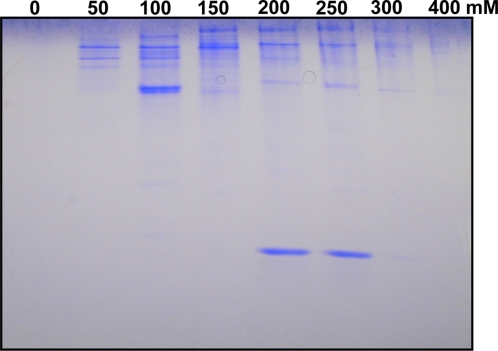
Separation of the fast migrating band isolated from polygyne workers collected in Dallas, TX. A thorax extract from 1000 workers from a field-collected colony was separated on a DEAE column and eluted with increasing salt concentration: 0–400 mM NaCl. The target protein, recovered in the 200–250 mM NaCl fractions, was further purified by gel filtration.

Having determined that the two forms of the protein were products of the same gene, we prospected for the elusive GP-9b in other parts of the gel. Intrigued by our inability to identify the second protein in these polygyne colonies from Riverside, CA, we analyzed polygyne samples from Dallas, TX. The gel profiles obtained with extracts from Dallas and Riverside polygyne workers were indistinguishable (data not shown) and they mirrored the profile obtained with a laboratory colony maintained at Texas A & M University. We isolated the fast migrating band from Dallas colonies by ion-exchange chromatography (Fig. 5), purified fractions 200 and 250 mM NaCl by gel filtration (data not shown), analyzed pure samples by LC-ESI/MS and obtained the same results as described above for the Riverside samples. We then concluded that contrary to a previous report [4], the three polygyne samples from field and laboratory colonies from two different geographical locations did not express GP-9b.

### Isolation of an Antennae-Specific Chemosensory Protein

**Figure 6:**
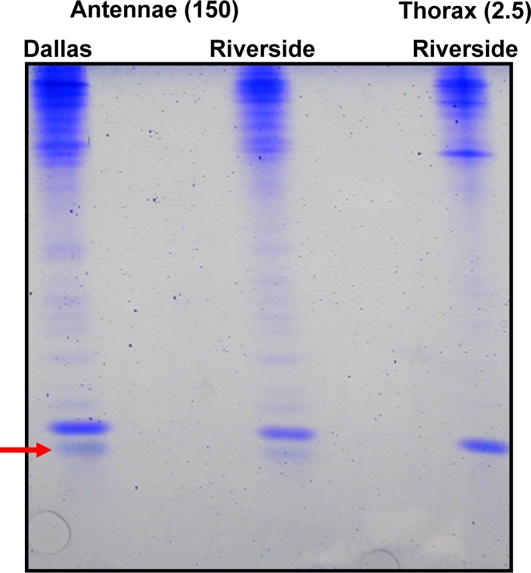
Comparison of protein extracts from antennae and thorax of polygyne workers. Antennal extracts from workers collected in Dallas and Riverside showed a band (highlighted with a red arrow), which migrated faster than GP-9B, and it was not present in extracts from thorax. Samples of 150 antennae-equivalent and 2.5 thoraces-equivalent were loaded in the gel.

**Figure 7:**
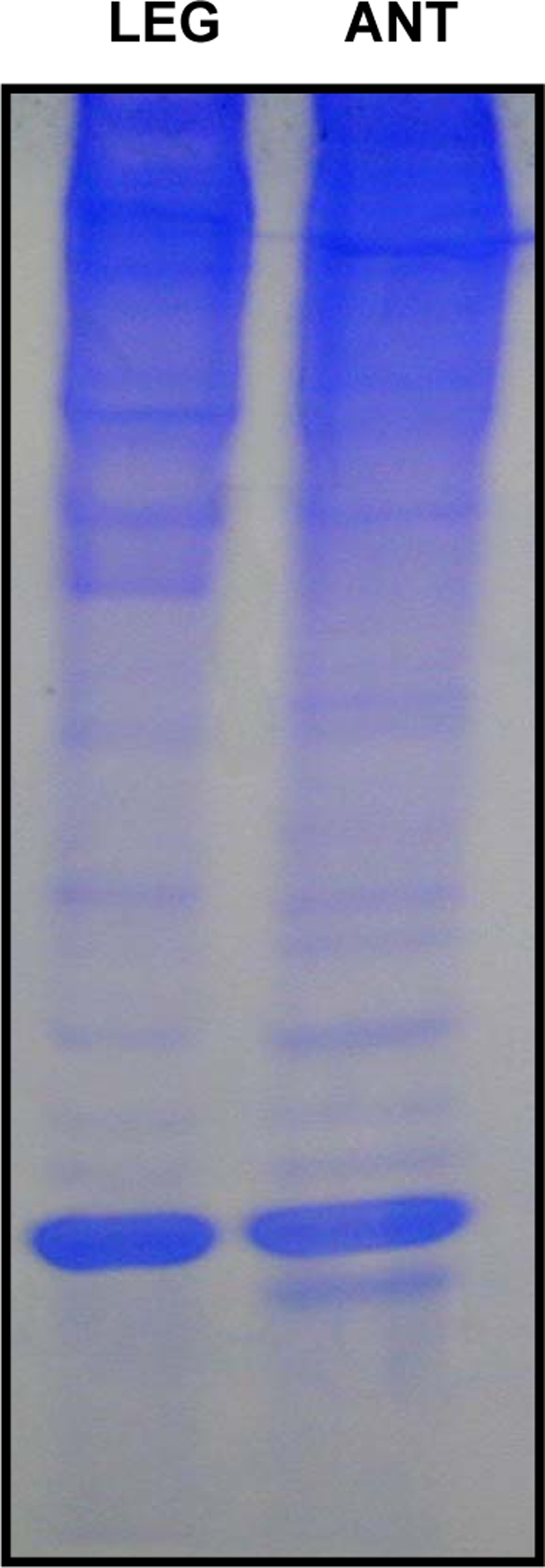
Analysis of extracts from olfactory and non-olfactory tissues. Extracts from legs (80 legs-equivalent) and antennae (200 antennae-equivalent) were separated on a 15% native gel. Bands above and below GP-9B were isolated from both tissues and sequenced. No traces of the band migrating just below GP-9B in the antennal extract were found in extracts from legs. Samples were extracted from workers collected in Riverside and Dallas.

**Figure 8:**
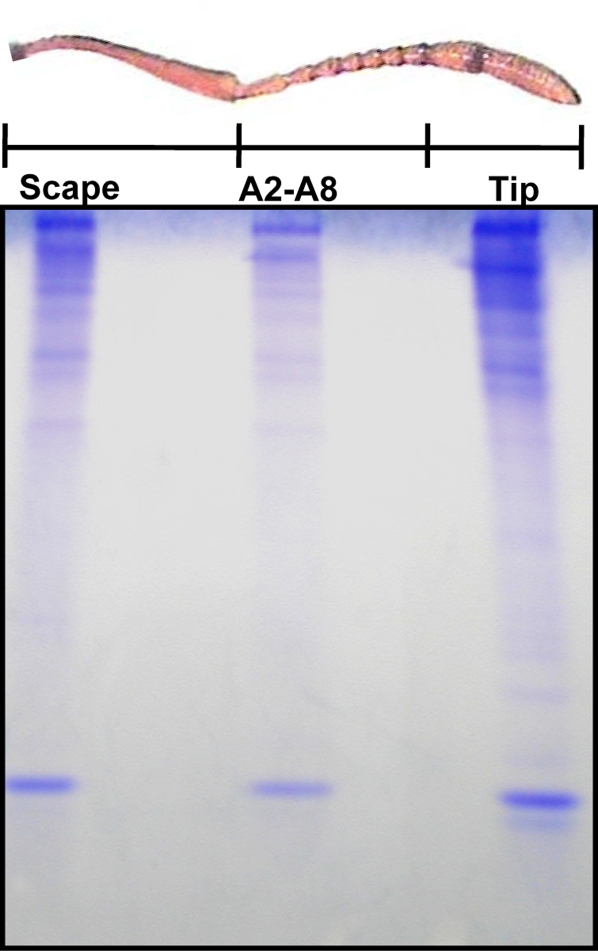
Expression of the antennae-specific protein is mapped to antennal club. The band of the antennae-specific protein was detected in the extracts from the club (A9 and A10 segments), but not in the intermediate (A2–A8) or basal (A1, scape) segments. Each lane was loaded with 100 antennae-equivalent. 18% Native-PAGE.

Although we could observe only one fast migrating band when analyzing samples from thorax and other non-olfactory tissues, extracts from antennae showed an even faster migrating band just below GP-9B (Fig. 6). This antennae-specific protein was not detected in the extracts from thorax extract (Fig. 6). Further comparison of extracts from olfactory and non-olfactory tissues showed that the antennae-specific band is not expressed in legs (Fig. 7). Initially, we have overlooked this fast migrating band when analyzing head extracts because it did not separate well from GP-9B in the high percentage (20%) gel (Fig. 2). To pinpoint the antennal tissues expressing this protein, we analyzed extracts from scape, intermediate segments (antennomeres A2–A8), and the terminal segments A9–A10 (club). Native PAGE analysis showed that the antennae-specific protein is expressed only in the club (Fig. 8), which is composed of the only segments on the antennae of workers housing sensilla trichodea curvata [14]. Therefore, olfactory proteins are expected to be expressed specifically in these segments.

**Figure 9:**
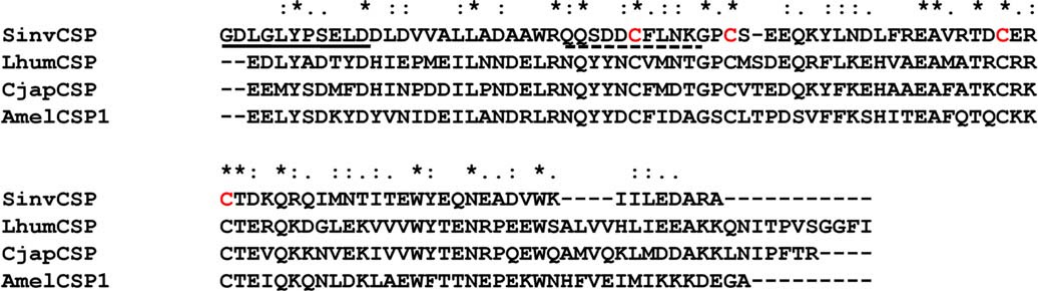
ClustalW alignment of the antennae-specific protein isolated from RIFA (SinvCSP) and other CSPs. Like other members of this family, SinvCSP possess 4 well-conserved cysteine residues (highlighted in red). Underline and dashed underline indicate amino acid sequences obtained by Edman degradation and tandem mass spectrometry, respectively. Asterisks, colons and dots show identity, higher and lower similarity at amino acid level, respectively. LhumCSP, *Linepithema humile* CSP (AY129672). CjapCSP, *Camponotus japonicus* CSP (Acc#, AB182637). AmelCSP1, *Apis mellifera* CSP1 (Acc#, DQ855482).

The N-terminal sequence Gly-Asp-Leu-Gly-Leu-Tyr-Pro-Ser-Glu-Leu-Asp was obtained by microsequencing the isolated antennae-specific protein. Attempts to clone the cDNA by using oligo-dT primer and a degenerate primer designed on the basis of N-terminal amino acid sequence were unsuccessful. Thus we digested this small antennae-specific protein with trypsin and obtained the internal sequence Gln-Gln-Ser-Asp-Asp-Cys-Phe-Leu-Asn-Lys, which was used for designing a second degenerate primer. Using the degenerate primers of RIFA-N-ASPf and RIFA-I-ASPr, we amplified weak and smear bands of 300 and 100 bp-long. The longer PCR product was a genomic fragment, whereas the 100 bp PCR product encoded amino acid sequences identified by mass spectrometry in the digestion products of the isolated protein. With gene-specific primers based on this 100 bp fragment, we cloned the whole sequence of the antennae-specific protein. With 13 independent clones we obtained the same cDNA. It consists of 403 bp and encoded 114 amino acid residues including a 22-amino acid-long signal peptide (Accession number AY713302). In addition to the internal sequences described above, the predicted amino acid sequence possessed amino acid sequences of peptides observed by mass spectrometry analysis of the digested native protein. These findings indicate that the cloned cDNA indeed encodes the isolated antennae-specific protein. The mature antennae-specific protein including 92 amino acid residues possessed 4 cysteine residues. The calculated molecular mass and isoelectric point were 10,702 Da and pI 4.19, respectively. FASTA revealed that this mature antennae-specific protein had 32.1, 30.6, and 30.6% identities with *Linepithema humile* chemosensory protein (CSP) (Acc#, AY129672), *Camponotus japonicus* CSP (Acc#, AB182637), *Apis mellifera* CSP1 (Acc#, DQ855482), respectively (Fig. 9). We, therefore, named the antennae-specific protein *S. invicta* chemosensory protein, SinvCSP.

### GP-9s are Hemolymph Proteins

**Figure 10:**
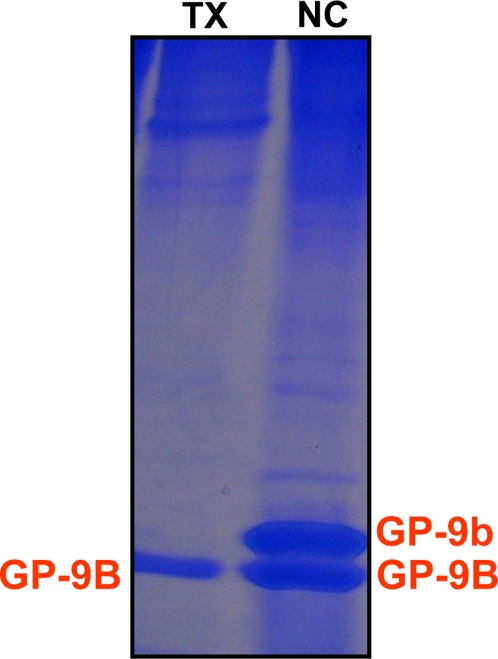
Polygyne forms of the red imported fire ant showing two types of GP-9 profiles. 18% Native PAGE analysis of thorax extracts from polygene queens from a laboratory colony (NC, North Carolina) and field collected (TX, Dallas) (ca. 1 queen-equivalent per lane). GP-9b is expressed in polygyne queens from North Carolina, but not in polygyne queens collected in Dallas, whereas GP-9B was detected in both samples. Similar pattern was observed with extracts from workers.

**Figure 11:**
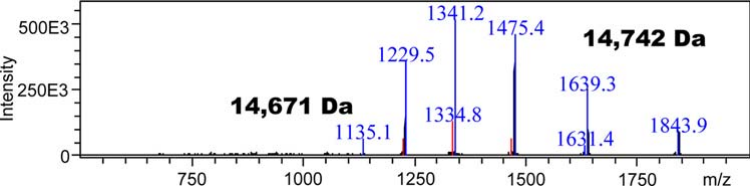
Mass spectrometer analyses of a sample of GP-9b isolated from workers and purified by chromatography. The charge envelope of the major protein was deconvoluted to give the molecular mass 14,742 Da, which was expected for GP-9b considering the formation of three disulfide bridges. The minor protein (14,671 Da) was not found in further purified samples.

Having being unable to isolate GP-9b from multiple polygyne colonies from two different geographical origins, we tried a fourth sample from North Carolina State University. Comparison of thorax extracts from queens of Dallas and North Carolina showed an outstanding band with slower migration than GP-9B (Fig. 10). Extracts from workers showed a similar profile (data not shown). We obtained a long N-terminal sequence of the band above GP-9B, Ser-Arg-Asp-Ser-Ala-Arg-Lys-Ile-Gly-Ser-Gln-Tyr-Asp-Asn-Tyr-Ala-Thr-Xxx-Leu-Thr-Glu-His-Gly-Leu. This allowed us to tentatively assign that band as GP-9b. Of notice, the N-terminal sequences of GP-9B and GP-9b differ in residues 20 (GP-9B, Ala; GP-9b Thr) and 23 (GP-9B, Ser; GP-9b, Gly). We obtained a larger sample of extract from workers, which was purified by ion-exchange chromatography and gel filtration, and analyzed by LC-ESI/MS. The first attempt to obtain a MS showed two proteins co-migrating (Fig. 11), with the major protein giving a MS profile expected for GP-9b (observed, 14,742 Da; calculated, 14,748 Da; 14,742 Da with formation of three disulfide linkages). The minor protein with molecular mass 14,671 seems to be unrelated to GP-9s and could be eliminated with collection of smaller fraction during gel filtration purification (data not shown). We sequenced 9 independent clones of the cDNA encoding this protein and obtained the same sequence of GP-9b that has been reported in the literature [4]. We analyzed antennal extracts from North Carolina workers and found three fast migrating bands, GP-9b, GP-9B, and SinvCSP (data not shown). Thus, SinvCSP was specifically expressed in the all polygyne workers investigated.

**Figure 12:**
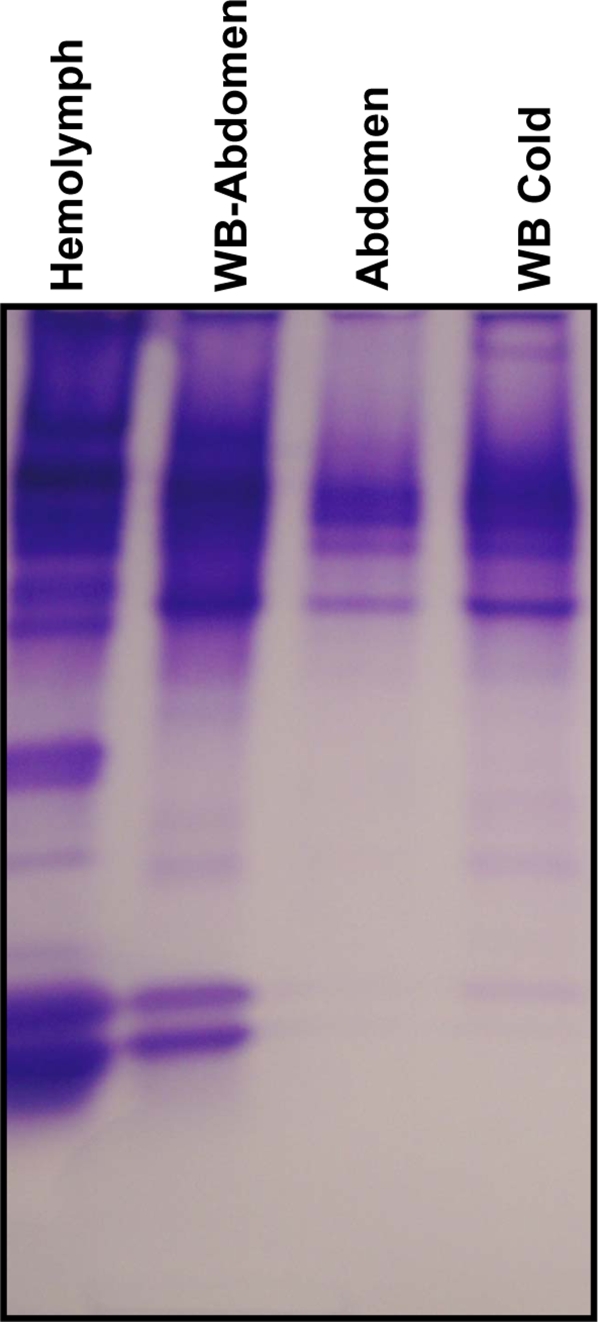
GP-9s are hemolymph proteins. High titers of GP-9B and GP-9b were observed in hemolymph extracts from workers from North Carolina, but these proteins are hardly detected in whole-body extracts. GP-9s are detected by extraction of the whole body after removal of the abdomen. Traces of these proteins were detected when the whole body was extracted at 4°C.

**Figure 13:**
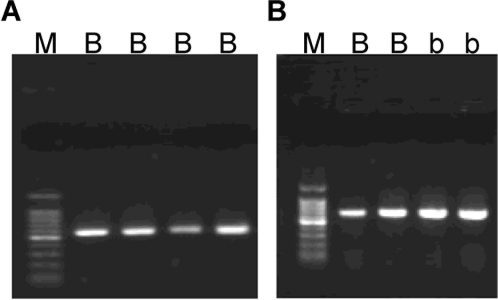
Confirmation of Gp-9s gene expression by RT-PCR and DNA sequencing. First strand cDNA was synthesized from polygyne fire ants collected in Riverside, CA (panel A) and a laboratory colony from North Carolina State University (panel B). Gp-9 cDNA fragments were amplified by RT-PCR (see Materials and Methods). The insert in pBluescript SK(+) was checked by PCR amplification with T3 and T7 promoter primers. Identity of the Gp-9 cDNAs, determined by DNA sequencing, is shown on top of each lane: B, Gp-9B cDNA; b, Gp-9b cDNA; M, DNA marker. RT-PCR data obtained with field and laboratory samples from Texas (data not shown) were identical to those with Riverside sample (A), i.e., no Gp-9b was detected.

Next, we examined the source of GP-9s. Native PAGE analysis of the extracts from hemolymph, whole body without abdomen, abdomen and whole body extracted at low temperature showed that a very high titer of both GP-9B and GP-9b in the hemolymph (Fig. 12). Because we did not detect these proteins in extracts from the abdomen we hypothesized that proteolytic enzymes from the midgut might degrade these hemolymph proteins. Degradation seems to be decreased when whole body were extracted and maintained at low temperature or the source of enzymes (abdomen) was removed (Fig. 12). Lastly, we have analyzed all samples by RT-PCR, isolated the bands and obtained full cDNA sequences. We found Gp-9B in all samples, but Gp-9b cDNA was obtained only from the polygyne sample from North Carolina (Fig. 13). These gene expression data mirror protein expression, and confirmed that polygyne samples from Riverside and Texas were devoid of Gp-9b.

In conclusion, it is highly unlikely that GP-9s are involved in olfactory mediation of social organization of the red imported fire ant. First, our data do not support the earlier hypothesis that both GP-9B and GP-9b are expressed in ants of polygyne colonies [4]. We have evaluated 4 polygyne colonies and only one of these samples, a lab colony from North Carolina, expressed GP-9b. We did not find GP-9b in polygyne workers from Riverside, CA, Dallas, TX, and a lab colony from Texas A&M University. In addition, our data strongly suggest that GP-9s are general hemolymph proteins found in all body parts. Sensory proteins are compartmentalized in the sensillar cavity and are isolated from hemolymph proteins that circulate in the hemocoel [15]. To the best of our knowledge there are no pheromone-binding proteins with demonstrated function which are expressed in other than olfactory tissues. There are, however, examples in the literature of proteins assigned as putative PBPs merely on the basis of their amino acid sequences, which are also expressed in non-olfactory tissues. There are multiple criteria for classification of proteins as OBPs [12], including but not limited to olfactory tissue specificity. Like an abundant 12.4 KDa hemolymph protein from the mealworm beetle, *Tenebrio molitor*, (THP12) [10], [11], GP-9B is a helical-rich protein. Therefore, GP-9s are more likely involved in the transport of lipids or small ligands in the hemolymph, with phosphorylation possibly serving as a molecular switch for controlling binding and release of ligands. If the expression of colony social organization in *S. invicta* is mediated by a queen-produced recognition primer pheromone [6], SinvCSP is more likely to be involved in the reception of this (these) semiochemical(s). As it has been demonstrated for a chemosensory protein from the Argentine ant, *Linepithema humile,* LhumCSP [16], SinvCSP is antennae-specific, and expressed in the last two segments housing olfactory sensilla. This is a testable hypothesis once the recognition primer pheromone is identified opening the door for binding assays, biochemical and sensory physiology studies.

## Materials and Methods

### Protein identification

Insect samples were collected from fields in Dallas, TX and Riverside, CA, and kindly provided by Drs. Kevin Heinz and Agenor Mafra Neto, respectively. Polygyny, the presence of multiple queens per nest, was confirmed at the time of the field collections. Thus, this queens and about 12% of the workers of this social form of *S. invicta* were considered to be heterozygous for the alleles encoding the general protein-9s Gp-9B and GP-9b [17]. Ants from laboratory colonies maintained at Texas A&M University and North Carolina State University were gifts from Drs. Kevin Heinz and Edward Vargo, respectively. Workers, males and queens were collected, immediately frozen, and transferred to our lab with dry ice and stored at −80°C until use. Antennae, heads, forelegs, midlegs, hindlegs, thoraces, and abdomens of worker (in a few cases thoraces from queens) were manually collected with clean fine forceps under a microscope. Hemolymph of workers was collected with glass capillary after puncturing with a fine needle through intersegmental membrane in the abdomen. To map the antennal tissues expressing an antennae-specific protein we meticulously collected separately: the tip of the antennae or club (9A and 10A segments; terminology according to Renthal et al., [14]), middle segment (flagellum except two prominent segments) and scape. In some cases the head-thorax complex and abdomen were collected separately. Collected samples were homogenized in 10 mM Tris-HCl, pH 8 with ice-cold Dounce tissue grinder (Wheaton, MillVille, NJ) and centrifuged twice at 12,000×g for 10 min. Supernatant was concentrated to appropriate volume with vacuum concentrator, separated with 15, 18 and 20% native PAGE and subsequently stained with Coomassie Brilliant Blue R-250 (CBB) (Bio-Rad, Hercules, CA).

### Protein purification and characterization

Antennae and other tissues were extracted in batches of 50–200 or 500–1000 ant-equivalent for analytical comparison by native PAGE and protein isolation, respectively. Queen extracts were obtained with 4–8 queens per extract. GP-9B, GP-9b and an antennae-specific protein (later named SinvCSP) were isolated by ion exchange (TSK gel DEAE Toyopearl 650S, Tosoh, Japan) eluting with 0, 50, 100, 150, 200, 250, 300, and 400 mM NaCl in 10 mM Tris·HCl, pH 8) and gel-filtration (Superdex 75, 16/60, GE Healthcare, Piscataway, NJ) with 150 mM NaCl in 20 mM mM Tris·HCl, pH 8). To determine N-terminal amino acid sequence, each purified protein was analyzed by native PAGE and transferred onto PVDF membrane prior to sequencing by Edman degradation at the UC Davis Molecular Structure Facility. To determine internal sequence protein bands were excised from gels, digested with trypsin, and the fragmented peptides were analyzed with a PE Sciex QSTAR Hybrid Quadrupole-TOF Mass Spectrometer (Applied Biosystems, Foster City, CA). Molecular mass was determined by LC-ESI/MS (LCMS-2010 Liquid Chromatograph Mass Spectrometer, Shimadzu, Japan). HPLC separations were done on a ZorbaxCB C8 column (150×2.1 mm, 5 µm; Agilent technologies, Santa Clara, CA) with a gradient of water and acetonitrile plus 2% acetic acid as modifier. The detector was operated with the nebulizer gas flow at 1 liter/min and the curve desolvation line and heat block at 250°C.

To identify a phosphorylated residue in GP-9B, an aliquot (200 µl) of the protein isolated by ion-exchange and gel filtration chromatography (see above) was dried in a SpeedVac and incubated with 25 µl of 8 M urea in 0.4 M ammonium bicarbonate, 2.3 µl of 100 mM DTT at 50°C for 15 min. The mixture was then treated with 5 µl of 100 mM iodoacetamide at room temperature for 15 min, diluted with 50 µl of water and 5 µl of acetonitrile, and analyzed by LC-ESI/MS. The treated protein was digested with 5 µl of Endoproteinase Lys-C (Boehringer Mannheim, Germany) at 37°C for 24 h. One aliquot was analyzed by LC-ESI/MS and the remainder was fractionated by HPLC (Agilent's 1100 Series) equipped with a ZorbaxCB C8 column and using water and acetonitrile with 0.1% trifluoroacetic acid at 0.3 ml/min and a gradient from 0–60% in 30 min. Fractions were collected manually every 0.5 min or when a peak was observed. Selected fractions were concentrated and analyzed by LC-ESI/MS, MALDI-TOF, and Edman degradation. To dephosphorylate GP-9B, an aliquot (50 µl) of the isolated protein sample (see above) was analyzed by LC-ESI/MS before and after incubation with 1 unit of alkaline phosphatase (Sigma, St. Louis, MO) for 2 h at room temperature.

CD spectra were recorded with a Jasco J-810 spectropolarimeter (Easton, MD) with isolated GP-9B (≈0.05 mg/ml) in 20 mM ammonium acetate, pH 7.

### cDNA cloning

Total RNA was extracted from 1,000 antennae and 10 thoraces of workers or queens with TRIzol Reagent (Invitrogen, Carlsbad, CA) after addition of glycogen (Roche Applied Science, Indianapolis, IN). First strand cDNA was synthesized from 1 µg of total RNA by SMART RACE cDNA Amplification Kit (Clontech, Mountain View, CA) and SuperScript II Reverse Transcriptase (Invitrogen) as a transcriptase. Synthesized cDNAs were treated with RNase H (Roche Applied Science). For PCR based-cDNA cloning of GP-9 cDNAs, we designed following gene specific primers according to Krieger and Ross (2002) and accession number AF459414 of genomic sequence: RIFA-GP-9f [5′-ATGAAGACGTTCGTATTGCATATTTTTATT-3′ (designed from signal sequence of Gp-9)]; RIFA-GP-9Br [5′-AATGGTTGTATGCCAGCTGTTTTTAATTGC-3′ (designed from 3′-untranslated region of *Gp-9B* allele genomic sequence)]; RIFA-GP9br [5′-TGCAGCTTTGTTAGAATCGGCGAGCACAGC-3′ (designed on the basis of the C-terminal sequence of Gp-9b)]. *PfuTurbo* Hotstart DNA polymerase (Stratagene, La Jolla, CA), which is one of the highest fidelity polymerases, was selected as a Taq polymerase. Following PCR program was carried out to amplify cDNA encoding GP-9s: 95° for 2 min; 40 cycles of 95°C for 30 s, 59°C for 30 s; 72°C for 1 min; 72°C for 10 min. Amplified cDNA fragments were directly gel-purified, ligated into *Eco* RV recognition site of pBluescript SK(+) (Stratagene) by T4 DNA ligase (New England Biolabs, Ipswich, MA). Thirty-seven independent clones were sequenced on an automated DNA sequencing facility (Davis Sequencing) to confirm existence of two forms of GP-9 cDNA.

For cDNA cloning of antennae-specific protein, following degenerate primers were designed on the basis of N-terminal and internal amino acid sequences (GDLGLYPSELD and QQSDDCFLNK) determined by Edman degradation or tandem mass spectrometry: RIFA-N-ASPf [5′-GG(A/C/G/T)GA(C/T)CT(A/C/G/T)GG(A/C/G/T)TT(A/G)TA(C/T)CC-3′]; RIFA-I-ASPr [5′-(C/T)TT(A/G)TT(A/C/G/T)A(A/G)(A/G)AA(A/G)CA(A/G)TC(A/G)TC(A/C/G/T)(C/G)(A/T)(C/T)TG(C/T)TG-3′]. Seventy cycles of stepwise program (94°C for 1 min, 50°C for 2 min and 72°C for 3 min) were carried out. One hundred base pair-length of DNA fragment was subcloned into pBluescript SK (+). The amino acid sequence predicted from amplified cDNA fragment was identical to that determined by Edman degradation and tandem mass spectrometry (data not shown). Based on this sequence, a gene-specific primer, RIFA-ASP1 (5′-GTGGCACTTCTGGCAGATGCCGCTTGGCG-3′) was designed. Following PCR program was carried out using UPM (Clontech) as 3′-RACE primer and *PfuTurbo* Hotstart DNA polymerase as Taq polymerase: 95°C for 2 min; 40 cycles of 95°C for 30 s; 50°C for 30 s and 72°C for 1 min; 72°C for 10 min. Based on the obtained 350 bp-length of PCR product (data not shown), another gene-specific primer, RIFA-ASP-2 (5′-CTGCCAGAAGTGCCACAACATCGAGATC-3′), was designed. 5′-RACE was conducted by same program for 3′-RACE. According to the sequence data of 5′-RACE product the designed RIFA-ASP-3 (5′-CGCAGTCAGTTCTTACAGCCTCTCTG-3′) was used to confirm the sequence of 5′-RACE product. DNA sequences were determined from 13 independent clones.

## References

[1] Lennartz FE (1973). Modes of dispersal of *Solenopsis invicta* from Brazil into the continental United States–a study in spatial diffusion. [MSc Thesis].

[2] Taber SW (2000). Fire ants.

[3] Ross KG (1997). Multilocus evolution in fire ants: effects of selection, gene flow and recombination.. Genetics.

[4] Krieger MJB, Ross KG (2002). Identification of a major gene regulating complex social behavior.. Science.

[5] Fletcher DJ, Blum MS (1983). Regulation of Queen Number by Workers in Colonies of Social Insects.. Science.

[6] Vander Meer RK, Alonso LE (2002). Queen primer pheromone affects conspecific fire ant (*Solenopsis invicta*) aggression.. Behav Ecol Sociobiol.

[7] Shoemaker DD, III JTC, Ross KG (1992). Estimates of heterozygosity in two social insects using a large number of electrophoretic makers.. Heredity.

[8] Wojtasek H, Leal WS (1999). Conformational change in the pheromone-binding protein from *Bombyx mori* induced by pH and by interaction with membranes.. J Biol Chem.

[9] Sandler BH, Nikonova L, Leal WS, Clardy J (2000). Sexual attraction in the silkworm moth: structure of the pheromone-binding-protein-bombykol complex.. Chem Biol.

[10] Rothemund S, Liou Y-C, Davies PL, Krause E, Sonnichsen FD (1999). A new class of hexahelical insect proteins revealed as putative carriers of small hydrophobic ligands.. Structure.

[11] Rothemund S, Liou Y-C, Davies PL, Sonnichsen FD (1997). Backbone structure and dynamics of a hemolymph protein from the mealworm beetle Tenebrio molitor.. Biochemistry.

[12] Leal WS (2005). Pheromone reception.. Top Curr Chem.

[13] Leal WS, Blomquist GJ, Vogt RG (2003). Proteins that make sense.. Insect pheromone biochemistry and molecular biology.

[14] Renthal R, Velasquez D, Olmos D, Hampton J, Wergin WP (2003). Structure and distribution of antennal sensilla of the red imported fire ant.. Micron.

[15] Steinbrech RA, Eguchi E, Tominaga Y (1999). Olfactory Receptors.. Atlas of Arthropod Sensory Receptors Dynamic Morphology in Relation to Function.

[16] Ishida Y, Chiang V, Leal WS (2002). Protein that makes sense in the Argentine ant.. Naturwissenschaften.

[17] Gotzek D, Ross GR (2008). Experimental conversion of colony social organization in fire ants (*Solenopsis invicta*): Workers genotype manipulation in the absence of queen effects.. J Insect Behav.

